# De Novo Genome Assembly, Genomic Features, and Comparative Analysis of the Sawfly *Dentathalia scutellariae*

**DOI:** 10.3390/biology15030214

**Published:** 2026-01-23

**Authors:** Shasha Wang, Chang Liu, Yang Mei, Deqing Yang, Huiwen Pang, Fang Wang, Gongyin Ye, Qi Fang, Xinhai Ye, Yi Yang

**Affiliations:** 1 State Key Laboratory of Rice Biology and Breeding and Ministry of Agricultural and Rural Affairs Key Laboratory of Molecular Biology of Crop Pathogens and Insects, Institute of Insect Sciences, Zhejiang University, Hangzhou 310058, Chinafangqi@zju.edu.cn (Q.F.);; 2 College of Plant Protection, Jilin Agricultural University, Changchun 130118, China; 3 College of Advanced Agriculture Science, Zhejiang A&F University, Hangzhou 311300, China; 4 Xianghu Laboratory, Hangzhou 311231, China

**Keywords:** sawfly, *Dentathalia scutellariae*, genome assembly, comparative genomics, gene family

## Abstract

*Dentathalia scutellariae* (Hymenoptera: Athaliidae) is a major pest of *Scutellaria baicalensis*, a plant of significant economic and medicinal value. To date, no genomic resources have been available for this species, limiting research into its biology and control. Here, we reported a genome assembly of *D. scutellariae* with high accuracy and contiguity, sequenced by PacBio HiFi long-read and MGI-Seq short-read methods. The genome assembly is 157.00 Mb in length with a contig N50 of 4.04 Mb. The complete BUSCO score was 98.8%. The genome contained 14.73 Mb of repetitive elements, representing 9.38% of the total genome size. We predicted 14,904 protein-coding genes, of which 12,327 genes were annotated functionally. Gene family analysis of *D. scutellariae* revealed 422 expanded and 113 contracted gene families. Notably, genes within expanded families were significantly enriched in retinol metabolism and drug metabolism–cytochrome P450 pathways. We present the first high-quality genome assembly of *D. scutellariae*, which serves as a foundational genomic resource. This dataset will facilitate future studies on the molecular basis of *D. scutellariae*’s pest status, host adaptation, and the development of targeted control strategies.

## Simple Summary

In recent years, *Dentathalia scutellariae* has inflicted significant damage on the medicinal plant *Scutellaria baicalensis*, yet genomic resources for this species have been limited to its mitochondrial genome. To address this, we present a high-quality genome assembly using PacBio HiFi long-read and MGI-Seq short-read sequencing. The assembled genome spans 157.00 Mb with a contig N50 of 4.04 Mb, a BUSCO completeness score of 98.8%, 9.38% repetitive sequences, and 14,904 predicted protein-coding genes. Comparative genomic and gene family analyses revealed significant expansions and contractions, with expansions notably enriched in retinol metabolism and drug metabolism–cytochrome P450 pathways. This genome provides a valuable genetic foundation for understanding the biology of *D. scutellariae* and serves as a critical resource for developing targeted pest control strategies to mitigate its impact on *S. baicalensis* cultivation.

## 1. Introduction

The family Athaliidae comprises the genera *Athalia*, *Dentathalia*, and *Hypsathalia* [1,2]. As a typical oligophagous group, species within Athaliidae are generally specialized on different plant families, namely either on species in Brassicaceae, Lamiaceae, Crassulaceae and Plantaginaceae [3]. Among them, some sawflies have become notorious pests due to their specialization on economic crops or medicinal plants [3,4]. To date, whole-genome sequences of several *Athalia* species have been successfully deciphered [5,6,7], and relevant research has systematically explored core scientific issues such as the mechanisms of dietary differentiation and adaptive strategies to host plant defense compounds [3,8,9]. These studies have provided important theoretical support for revealing the coevolutionary relationships between phytophagous insects and their host plants.

In contrast to the abundant research on *Athalia*, studies on the genus *Dentathalia* remain limited, and genomic resources for this group are scarce. *Dentathalia scutellariae* is a representative species of the genus *Dentathalia* and also a specialist pest of *Scutellaria baicalensis* Georgi. (Lamiaceae), which is a traditional and valuable medicinal plant in China [2,10]. The larvae of this pest feed exclusively on the fruit pods and seeds of *S. baicalensis*, causing the pods to become empty and leading to significant seed loss. Previous studies have shown that some *Athalia* species have evolved specialized physiological mechanisms to cope with respective defensive glucosides, such as sequestration of these compounds in hemolymph and metabolization of glucosinolates via sulfation at the sugar moiety catalyzed by sulfotransferases [3,8,9,11]. The ability of *D. scutellariae* to break through the chemical defense barriers of *S. baicalensis* suggests that it possesses unique molecular adaptations. Therefore, this study aimed to generate the first nuclear genome of *D. scutellariae* to uncover the genetic basis of its host adaptation and population mechanisms, which remain completely unexplored.

Deciphering the mechanisms of species adaptation and identifying molecular targets for pest control relies on both genomic resources and comparative data mining [12,13]. Here, we aim to fill the genomic gap for the genus *Dentathalia* by sequencing, assembling, and annotating the first nuclear genome of *D. scutellariae*. We constructed a high-quality draft genome of 157.00 Mb (contig N50 = 4.04 Mb), which encodes 14,904 protein-coding genes. This resource will enable comparative genomic analyses to elucidate the phylogenetic evolution and dietary differentiation within Athaliidae. Furthermore, it will illuminate the molecular mechanisms underlying *D. scutellariae*’s adaptation to *S. baicalensis*, providing a theoretical foundation and molecular targets for developing precise, eco-friendly control strategies for this key medicinal plant pest.

## 2. Materials and Methods

### 2.1. Sample Preparation and Sequencing

*D. scutellariae* individuals were collected from a *S. baicalensis* cultivation field (40°59′36″ N, 117°24′2″ E) located in Chengde, Hebei Province, China. Following dissection to remove the gut and abdominal tissues, sawflies were immediately snap-frozen in liquid nitrogen and stored at −80 °C until subsequent experimental use. Genomic DNA was extracted from 24 adult individuals (23 females and 1 male) of *D. scutellariae* using the QIAGEN Genomic-tip Kit (QIAGEN, Paisley, UK). The quality of the extracted DNA was assessed using a NanoDrop ONE Microvolume Spectrophotometer (Thermo Fisher Scientific, Waltham, MA, USA), which showed optimal purity (A260/A280 = 1.81, A260/A230 = 2.01). DNA integrity was verified by agarose gel electrophoresis, revealing a high-molecular-weight band (>20 kb) with minimal degradation. The concentration was accurately quantified using a Qubit Fluorometer (Thermo Fisher Scientific, Waltham, MA, USA), yielding a total of approximately 6.5 µg of high-quality DNA. The Single Molecule Real Time (SMRT) libraries and 150 bp paired-end libraries were separately constructed using the SMRTbell Express Template Prep Kit 2.0 (PacBio, Menlo Park, CA, USA) and the TruSeq DNA Sample Prep Kit (Illumina, Inc., San Diego, CA, USA) according to the manufacturer’s instructions. Ultimately, the SMRT library was sequenced on the PacBio Revio platform, while the paired-end library was sequenced on the MGI T7 platform. All sequencing work was performed by Grandomics Biosciences Company (Wuhan, China).

### 2.2. Genome Features Assessment

Raw short-read sequencing data were first processed with fastp v0.23.4 [14] under default parameters to perform adapter trimming and filter out low-quality reads. Subsequently, the resulting high-quality clean reads were used to estimate the genome size, heterozygosity, and repeat content using GenomeScope2.0 [15] with a default k-mer length of 21.

### 2.3. Genome Assembly and Assessment

Whole-genome sequencing on the PacBio Revio platform yielded 7.02 Gb of high-quality PacBio HiFi long reads, which were used directly for subsequent assembly, given the exceptional raw data quality (98.99% of reads >10 kb). To determine the best initial assembly of *D. scutellariae*, we evaluated two software programs, NextDenovo v2.5.2 [16] and Hifiasm v0.24.0 [17]. Following a comparative assessment of continuity metrics (e.g., Contig N50), NextDenovo demonstrated superior performance and was consequently selected for de novo genome assembly using default parameters, which produced a primary haplotype assembly of 158.08 Mb with a Contig N50 of 4.04 Mb. Given the high base accuracy of the PacBio HiFi reads and the integrated correction functions of NextDenovo, no separate polishing step was applied.

The completeness of the *D. scutellariae* genome assembly was assessed using Benchmarking Universal Single-Copy Orthologs (BUSCO) v5.8.0 [18] against the insecta_odb12 database. Additionally, reference-free k-mer-based evaluation using MGI-seq short read and PacBio HiFi long read datasets (QV = 50.08 and 53.42, respectively; k-mer completeness >95%) further confirmed the high accuracy and completeness of the assembly [19,20]. Sequence consistency analysis showed alignment rates of 98.53% and 98.79% to the assembly [21,22,23,24]. Potential contaminant sequences were removed by aligning against the Nucleotide Sequence Database (NT), yielding a final high-quality genome of 157.00 Mb with a Contig N50 of 4.04 Mb.

Separately, the mitochondrial genome was assembled using MitoHiFi v3.0.0 [25], which incorporates MitoFinder v1.4.1 [26] for annotation and selection of the final mitochondrial contig, followed by manual inspection. The resulting mitogenome was annotated and visualized with OGDRAW v1.3.1 [27].

### 2.4. Genome Annotation

Repetitive sequences within the genome were identified following an established genome annotation pipeline (https://github.com/meiyang12/Genome-annotation-pipeline, accessed on 15 October 2025). Briefly, a custom reference repeat library (Insecta_ad.fa) was constructed using the famdb.py and buildRMLibFromEMBL.pl scripts from RepeatMasker v4.1 [28,29]. Subsequently, transposable elements, including LTR (Long Terminal Repeat), TIR (Terminal Inverted Repeat), Helitron, LINE (Long Interspersed Nuclear Element), and SINE (Short Interspersed Nuclear Element, were annotated using HiTE v3.3 [30]. For comparative analysis, the genomes of two related sawfly species, *Athalia rosae* (GCA_917208135.1) and *Athalia cordata* (GCA_963932425.1), were downloaded from the National Center for Biotechnology Information (NCBI) database and annotated for repetitive sequences using the same pipeline.

The repeat-masked genome was used for structural gene prediction with the BRAKER v3 pipeline [29,31]. Functional annotation of predicted protein-coding genes was performed by searching against databases Pfam and UniProt using HMMer v3.4 [32] or DIAMOND v2.1.14 [33]. Gene Ontology (GO) terms and Kyoto Encyclopedia of Genes and Genomes (KEGG) pathway assignments were generated with eggNOG-mapper v2.1.12 [34]. Additionally, non-coding RNA was identified in the repeat-masked genome using Infernal v1.1.5 [35] and Rfam database [36].

### 2.5. Comparative Genomics Analysis

Genomic data and corresponding annotation files for the analyzed insect species were obtained from public databases, including NCBI and InsectBase 2.0 (data acquired before 1 November 2025). Gene families were inferred from protein sequences using OrthoFinder v3.0.1b1 [37], which identified 2876 single-copy orthologous groups. Protein sequences from these orthogroups were aligned with MAFFT v7.505 [38], concatenated into supergenes, and trimmed following the OrthoFinder pipeline (https://github.com/davidemms/OrthoFinder, accessed on 3 November 2025). A maximum-likelihood (ML) phylogenetic tree was constructed using IQ-TREE v2.1.2 [39] with the best model (LG + I + G) selected by ModelFinder [40]. Species divergence times were subsequently estimated using r8s v1.81 [41] based on previously published studies [42,43,44]. The time points were as follows: Hymenoptera: 223.7 to 304.0 mya; Ichneumonoidea: 151 to 218 mya; Apoidea + Formicoidea: 100.3 to 163.5 mya.

To investigate gene family dynamics, we employed CAFE5 [45] to infer gene family expansions and contractions across each phylogenetic branch. Gene families exhibiting significant expansion (*p* < 0.05) along the branch leading to our target species were retained for further functional investigation. Finally, GO and KEGG enrichment analyses were conducted for these significantly expanded gene families using the R package clusterProfiler v4.10.0 [46].

## 3. Results

### 3.1. Genome Features Assessment and Assembly

**Figure 1:**
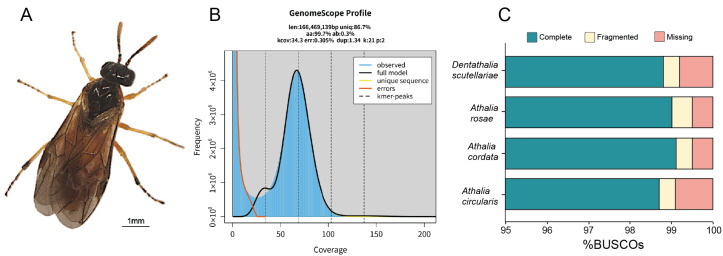
Assembly of the genome of *Dentathalia scutellariae*. (**A**) Dorsal view of an adult *D. scutellariae*. Scale bar: 1 mm. (**B**) K-mer analysis of *D. scutellariae* with GenomeScope 2 (K = 21). (**C**) Comparison of the completeness of genome assemblies.

The sequencing data for *D. scutellariae* genome assembly consisted of 7.02 Gb of PacBio HiFi long reads and 14.70 Gb of MGI-Seq short reads, amounting to 43.63× and 90.97× coverage of the whole genome, respectively. With k = 21, the estimated genome size of *D. scutellariae* was 166.5 Mb, with 0.3% heterozygosity and 1.34% repeat contents (Figure 1B).

We performed a de novo assembly of the PacBio HiFi long reads using NextDenovo, yielding a preliminary genome size of 158.08 Mb. After removing contaminant sequences, the final genome size was 157.00 Mb, comprising 128 contigs with a contig N50 of 4.04 Mb and a longest contig of 10.06 Mb, indicating high continuity. The GC content of the assembled genome was 36.95%. The assembly size is in strong agreement with a prior k-mer-based estimate of 166.5 Mb.

To evaluate genome completeness and accuracy, BUSCO analysis was performed against the insecta_odb12 dataset. Results showed that 98.8% of conserved orthologs were complete, of which 98.6% were single-copy and 0.2% duplicated; only 0.4% were fragmented and 0.8% missing (Figure 1C). These metrics confirm high gene-space completeness and contiguity, consistent with the observed contig-level assembly quality, and support the utility of this genome for downstream comparative and functional genomic investigations.

### 3.2. Repetitive Sequence Annotation

Genome repeat sequence analysis revealed that *D. scutellariae* and the other two sawfly species, *A. rosae* and *A. cordata*, all exhibit low repeat sequence content, with total proportions of 9.38%, 8.76%, and 8.13%, respectively (Table 1). Regarding the composition of repetitive elements, the proportions of interspersed repeats in the three species are 4.34%, 5.00%, and 4.04%, among which DNA transposons and LTR elements are the major repeat types (Table 1). Notably, simple repeats constitute the most dominant repeat type in all three species, accounting for 3.91%, 2.99%, and 3.07%, respectively (Table 1). Additionally, SINEs and LINEs were detected in *D. scutellariae* and *A. cordata* (Table 1). These results suggest a conserved, repeat-poor genomic architecture in the three analyzed sawfly species, with subtle lineage-specific variations.

### 3.3. Gene Annotation

**Figure 2:**
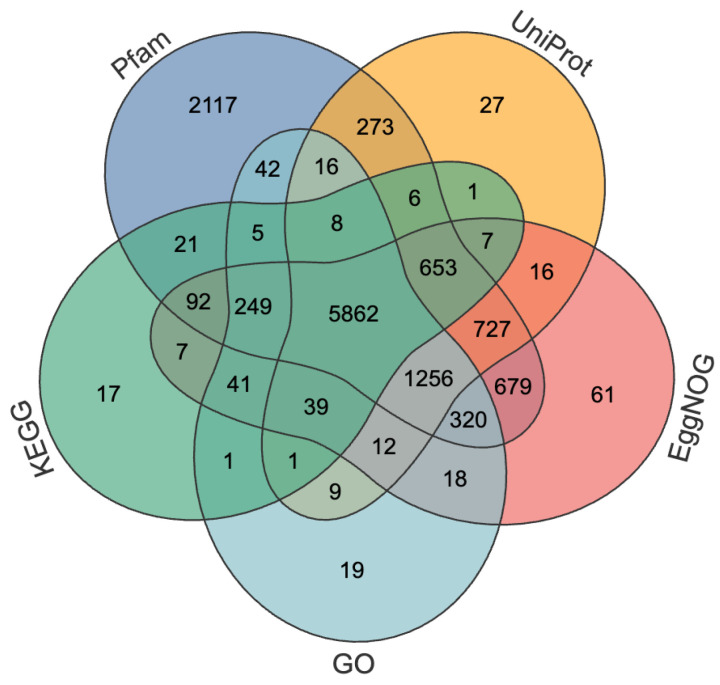
Venn diagram of functional annotations for the predicted protein-coding genes. The diagram illustrates the distribution and overlap of genes annotated with databases (e.g., Pfam, EggNOG). The different colors represent the different databases. The numerical values within each section indicate the count of genes uniquely assigned or shared between the annotation categories.

A total of 14,904 protein-coding genes, 169 tRNAs, 67 rRNAs, 61 miRNAs, and 51 snRNAs were identified. The mean length of genes was 3558.31 bp, and the mean length of coding sequence (CDS) was 1663.10 bp (Table 2). When compared to *A. rosae*, *D*. *scutellariae* has approximately 30.8% more protein-coding genes, yet its average gene length is less than half (41.6%) of that in *A. rosae* (Table 2). The average CDS lengths of the two species are relatively comparable (Table 2). The annotation ratios of the predicted genes in *D*. *scutellariae* were 47.03%, 52.99%, 59.80%, 67.36%, and 82.71% in KEGG, GO, UniProt, EggNOG, and Pfam databases, respectively (Figure 2).

### 3.4. Mitochondrial Genome Assembly

**Figure 3:**
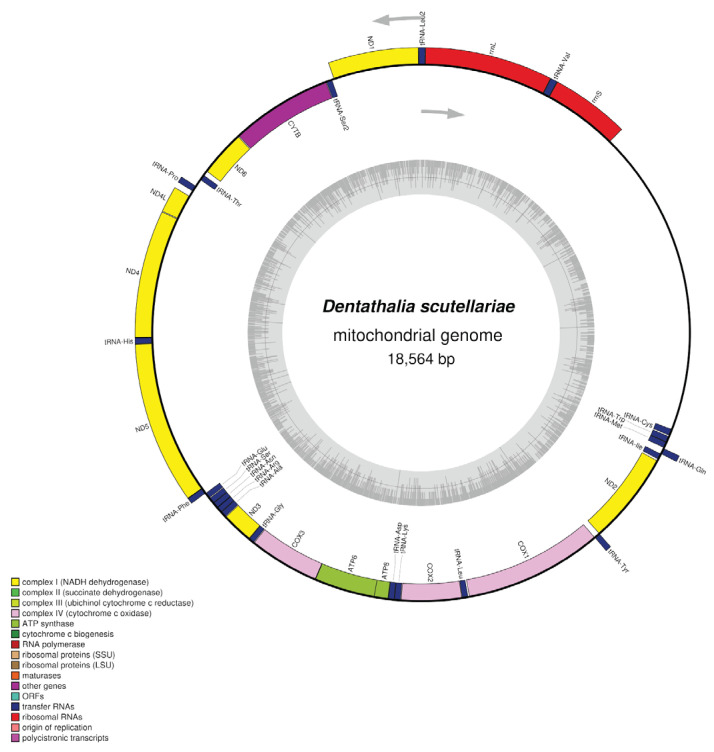
The circular mitochondrial genome map of *Dentathalia scutellariae*. The complete mitochondrial genome is 18,564 bp in length. Genes are represented by different colored blocks. The arrows indicate the transcription direction. The colored blocks outside each ring indicate that the genes are on the direct strand, while colored blocks within the ring indicate that the genes are located on the reverse strand. The innermost dark gray lines indicate GC content.

We further assembled the mitochondrial genome of this sawfly using PacBio HiFi long-read sequencing data. The results showed that the complete mitochondrial genome is 18,564 bp in length with a GC content of 20.24% (Figure 3). The mitochondrial genome annotation identified 13 protein-coding genes (PCGs): one ATP synthase subunit 8 (ATP8), one ATP synthase subunit 6 (ATP6), seven NADH dehydrogenase subunits (ND1, ND2, ND3, ND4, ND4L, ND5, ND6), three cytochrome c oxidase subunits (COX1, COX2, COX3), and one cytochrome b (CYTB), along with 22 transfer RNA (tRNA) genes and 2 ribosomal RNA (rRNA) genes (Figure 3). Alignment of the HiFi-assembled mitochondrial genome in this study with the previously published version assembled from Illumina data (NCBI Reference Sequence: NC_067793.1, 16,349 bp in length) [2] revealed a high sequence identity of 99.12%, confirming the genetic stability of the mitochondrial genome in this species. Furthermore, compared to the NGS-assembled version, the HiFi-assembled mitochondrial genome exhibits superior sequence completeness and accuracy in complex regions such as the terminal regions of the genome, providing a more reliable sequence reference for subsequent related studies.

### 3.5. Comparative Genomics

**Figure 4:**
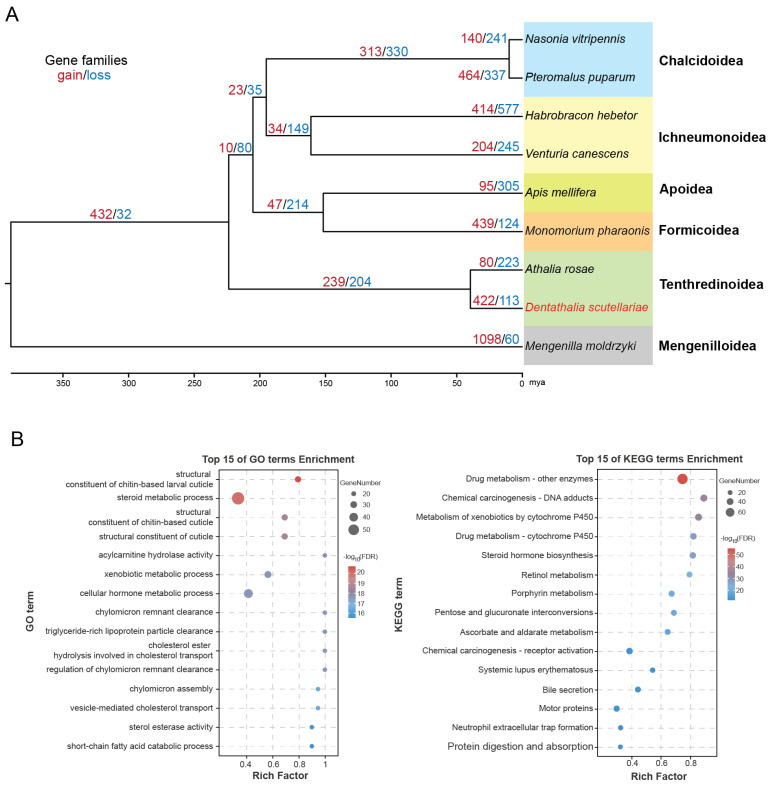
Phylogenetic and comparative genomic analysis of *Dentathalia scutellariae*. (**A**) The maximum likelihood phylogenetic tree built from 2876 concatenated single-copy orthologous groups from *D. scutellariae* and 8 other species using IQ-TREE. *Mengenilla moldrzyki* was used as an outgroup. All nodes received 100% bootstrap support. The colored background blocks highlight different superfamilies. The expansion numbers of gene families (red) and contraction (blue) are shown on the branches. (**B**) Enrichment of GO and KEGG terms with the significantly expanded gene families in *D. scutellariae*.

We investigated the evolutionary relationships among *D. scutellariae* and eight other insect species (*Mengenilla moldrzyki*, *A. rosae*, *Pteromalus puparum*, *Nasonia vitripennis*, *Habrobracon hebetor*, *Venturia canescens*, *Monomorium pharaonic*, *Apis mellifera*) using a phylogenetic tree constructed from 2876 single-copy orthologous genes (Figure 4A). The analysis revealed that *D. scutellariae* and *A. rosae* were most closely related, and both belong to the Tenthredinoidea superfamily. The members of the Chalcidoidea and Ichneumonoidea superfamily were found to be clustered together separately. Divergence time estimation based on this phylogeny indicated that *D. scutellariae* and *A. rosae* diverged approximately 39.6 million years ago (Figure 4A).

Gene family evolution analysis revealed that *D. scutellariae* has experienced significant genomic changes, with 422 expanded and 113 contracted gene families (Figure 4A). To explore the biological functions of these expansions, we performed GO and KEGG enrichment analyses on the 154 significantly expanded gene families (comprising 1181 genes; *p* < 0.05; Table S1). The GO analysis highlighted strong enrichment in functional categories related to xenobiotic detoxification, cuticle remodeling, lipid metabolism, and hormone regulation (Figure 4B), suggesting genomic adaptations linked to its specialized diet. The KEGG analysis further corroborated these findings, revealing significant enrichment in three major pathways: xenobiotic metabolism, endogenous hormone and lipid metabolism, and energy supply (Figure 4B), which collectively underpin *D. scutellariae*’s ability to overcome the chemical defenses of its host plant, *S. baicalensis*.

## 4. Discussion

In this study, we report the genome sequence of *D. scutellariae* containing 9.38% repetitive content and 14,904 annotated protein-coding genes. A comparison of contig-level genome assembly revealed a better contiguity of the *D. scutellariae* genome as compared to the previously reported cephid sawfly (*Cephus spinipes*) and figwort sawfly (*Tenthredo scrophulariae*) genomes [47,48]. Furthermore, the number of annotated protein-coding genes in *D. scutellariae* is approximately 30.8% higher than that in the *A. rosae* genome [6]. This difference could be attributed to lineage-specific gene family expansions (Figure 4A) and/or to methodological differences in gene prediction and annotation. Notably, the proportion of repetitive sequences in *D. scutellariae* is lower than that reported in other hymenopteran insects (e.g., *Theocolax elegans*, 56.4%) [49] but consistent with that of congeneric species in the family Athaliidae (Table 1). This relatively low repetitive content may indicate a simplified genome structure characteristic of Athaliidae species.

Phylogenetic analysis revealed a close evolutionary relationship between *D. scutellariae* and *A. rosae*, with an estimated divergence time of ~39.62 million years ago (Figure 4A). Although both species belong to the family Athaliidae, their larvae exhibit distinct ecological niche differentiation in feeding behavior [2]: *A. rosae* feeds on Brassicaceae plants and sequesters glucosinolates in its hemolymph as a defensive strategy [3,9], whereas *D. scutellariae* specializes in feeding on the fruits of the medicinal plant *S. baicalensis*, which possesses a unique chemical defense system dominated by flavonoids and their glycosides [10]. Flavonoids can reduce the survival and growth of the European corn borer [50] and decrease both larval weight and the development time of *Mamestra configurata* larvae and pupae [51]. Flavonoids from *Ginkgo biloba* exhibit significant anti-feeding activity against *Hyphantria cunea* [52].

Herbivorous insects have evolved diverse adaptive strategies to cope with plant defensive compounds. For instance, some species sequester these compounds into their cuticle to enhance protection against pathogens and predators, or into their wings to facilitate mate attraction [53,54]. In *D. scutellariae*, significantly expanded gene families were enriched in functional categories related to “structural constituent of chitin-based larval cuticle” and “structural constituent of cuticle” (Figure 4B). These GO terms may suggest a potential physical sequestration mechanism, wherein flavonoids are retained within the cuticle to reduce their internal toxicity, while also reinforcing cuticle integrity as a mechanical barrier, potentially in response to feeding on tough plant tissues or as a general defense against plant surface compounds. This interpretation, inferred from genomic enrichment patterns, provides a testable hypothesis for future experimental validation.

Furthermore, insects utilize detoxification enzymes, such as cytochrome P450s, carboxylesterase (CarE), and glutathione S-transferase (GST) to metabolize and detoxify plant secondary metabolites like flavonoid allelochemicals [55]. For example, the honeybee P450 CYP6AS can metabolize the flavonoid quercetin [56]. *Aoria nigripes* relies on detoxification and protective enzyme systems, including cytochrome P450, carboxylesterase, and peroxidase, to mitigate the adverse effects of high flavonoid levels in its host plant [57]. In *Helicoverpa zea*, at least two specific P450s, CYP6B8 and CYP321A1, are associated with detoxification of flavone, another flavonoid [58,59]. Similarly, in *Helicoverpa armigera*, differentially expressed genes induced by flavone are primarily concentrated in retinol metabolism and drug metabolism–cytochrome P450 pathways [60]. Consistent with these findings, expanded gene families in *D. scutellariae* were also significantly enriched in retinol metabolism and drug metabolism–cytochrome P450 pathways (Figure 4B), indicating the evolution of efficient metabolic detoxification as a key adaptive response to the flavonoid-rich *S. baicalensis*.

In summary, this study reveals that *D. scutellariae* may have evolved an integrated adaptive strategy to cope with its flavonoid-rich host, *S. baicalensis*, which involves physically sequestering flavonoids to reduce internal exposure and toxic load while also constructing efficient metabolic detoxification capabilities through the extensive expansion of cytochrome P450 gene families. Although the retinol metabolism pathway can be regulated in insects upon exposure to xenobiotics such as flavonoids [60,61,62], its precise functional role in *D. scutellariae* requires further experimental investigation. These findings help explain how insects achieve chemical adaptation to specialized hosts and provide an important theoretical foundation for subsequent identification of key detoxification P450 genes in *D. scutellariae*, as well as for elucidating their functions and regulatory networks.

## 5. Conclusions

In conclusion, this study presents the first nuclear genome assembly for the genus *Dentathalia*, specifically for *D. scutellariae*. The assembly demonstrates high accuracy and contiguity, providing a foundational genomic resource for the family Athaliidae. Comprehensive annotation and comparative analyses revealed distinctive genomic features, including gene family expansions potentially linked to cuticle development and detoxification. This resource not only advances our understanding of sawfly evolution and biology but also enables precise comparative genomics within Athaliidae. It will facilitate future studies on insect–plant coevolution, support the development of targeted pest management strategies, and contribute to broader investigations into hymenopteran genome evolution.

## Figures and Tables

**Table 1:** Repetitive elements found by RepeatMasker.

Element Type	*Dentathalia scutellariae*			*Athalia rosae*			*Athalia cordata*		
	Num ^1^	LO (bp) ^2^	PS ^3^	Num	LO (bp)	PS	Num	LO (bp)	PS
Total interspersed repeats	-	6,819,110	4.34%	-	8,605,934	5.00%	-	6,828,764	4.04%
SINEs	250	23,817	0.02%	0	0	0.00%	49	9578	0.01%
LINEs	3407	267,248	0.17%	0	0	0.00%	72	63,300	0.04%
LTR elements	3940	2,188,477	1.39%	5666	2,762,375	1.61%	1414	1,877,030	1.11%
DNA transposons	18,871	3,620,076	2.31%	20,299	5,843,559	3.40%	12,717	4,878,856	2.89%
Unclassified	7034	719,492	0.46%	0	0	0.00%	0	0	0.00%
Small RNA	329	61,958	0.04%	0	0	0.00%	49	9578	0.01%
Satellites	1	79	0.00%	0	0	0.00%	0	0	0.00%
Simple repeats	156,653	6,140,166	3.91%	129,325	5,133,883	2.99%	123,472	5,184,289	3.07%
Low complexity	33,557	1,607,487	1.02%	26,068	1,315,192	0.76%	29,831	1,445,383	0.85%

^1^ Number of elements. ^2^ Length occupied in base pairs. ^3^ Percentage of element type with regard to the assembled genome sequence.

**Table 2:** Comparative annotation features of *Dentathalia scutellariae* and *Athalia rosae*.

Feature	*Dentathalia scutellariae*	*Athalia rosae* [6]
Protein-coding genes	14,904	11,393
BUSCO (%) (Annotation)	C ^1^: 98.4	C: 99.3
Average gene length (bp ^2^)	3558.31	8560.96
Average number of exons per transcript	6.11	6.78
Average CDS ^3^ length (bp)	1663.10	1767.84

^1^ BUSCO (Benchmarking Universal Single-Copy Orthologs) completeness assessment was performed using the insecta_odb12 database, with results presented as the percentage of complete orthologs (C%). ^2^ Base pairs. ^3^ Coding sequence.

## Data Availability

The raw sequencing data generated in this study have been deposited in the Genome Sequence Archive in National Genomics Data Center, China National Center for Bioinformation/Beijing Institute of Genomics, Chinese Academy of Sciences (GSA: CRA037257) that are publicly accessible at https://ngdc.cncb.ac.cn/gsa (accessed on 20 January 2026).

## Supplementary Materials

The following supporting information can be downloaded at https://www.mdpi.com/article/10.3390/biology15030214/s1. Table S1: Gene families with significant expansion in *Dentathalia scutellariae* (*p* < 0.05).
